# Antibacterial activity and physicochemical properties of light-curable fluoride varnishes containing strontium phosphate-based glass

**DOI:** 10.1007/s00784-025-06612-x

**Published:** 2025-10-20

**Authors:** Na-Yeon Kim, Mi-Sol Ryu, Ji-Min Lee, Soo-Yeon Jeong, Hye-Been Choi, Myung-Jin Lee, Song-Yi Yang

**Affiliations:** 1 Department of Dental Hygiene, Konyang University, 158 Gwanjeodong-ro, Seo-gu, Daejeon, 35365 Republic of Korea; 2 Department of Dental Hygiene, Division of Health Science, Baekseok University, Cheonan, 31065 Chungcheongnam-do Republic of Korea

**Keywords:** Antibacterial activity, Light-curable fluoride varnish, Physicochemical properties, Strontium phosphate-based glass

## Abstract

**Objectives:**

Light-curable fluoride varnishes (LCFVs) have demonstrated enhanced long-term protection of the enamel compared to conventional fluoride varnishes. Strontium-containing phosphate-based glass (Sr-PBG) exhibits antibacterial activity and promotes remineralization through sustained release of calcium, phosphate, and strontium ions. This study aimed to evaluate the antibacterial efficacy and physicochemical properties of LCFVs containing various concentrations of Sr-PBG.

**Methods:**

Experimental LCFVs were formulated by incorporating Sr-PBG powder at concentrations of 0 (control), 1.0, 5.0, and 10.0 wt%. Antibacterial effects were assessed by colony-forming units (CFU) of *Streptococcus mutans* (*S. mutans)* after 24 and 48 h of incubation, and bacterial adhesion was examined by scanning electron microscopy (SEM). The physicochemical properties, including the degree of conversion, film thickness, contact angle, SEM, and ion release, were evaluated. Data were analyzed using one-way analysis of variance and Tukey’s post-hoc tests (α = 0.05).

**Results:**

All Sr-PBG groups exhibited significantly reduced *S. mutans* CFU compared to the control (*p* < 0.05), with antibacterial effects enhanced by longer incubation. Sr-PBG incorporation did not significantly alter the degree of conversion or film thickness (*p* > 0.05). The contact angle significantly decreased only for 5.0 wt% Sr-PBG (*p* < 0.05). SEM analysis showed slight particle agglomeration at higher Sr-PBG concentrations, whereas the ion release of Sr and P significantly increased (*p* < 0.05).

**Conclusions:**

Incorporating ≥ 1.0 wt% Sr-PBG into LCFVs significantly enhanced antibacterial activity against *S. mutans* without compromising physicochemical properties.

**Clinical relevance:**

Sr-PBG-containing LCFVs may offer improved clinical efficacy for caries prevention compared with conventional fluoride varnishes, providing enhanced antibacterial performance and promoting an environment favorable for remineralization.

## Introduction

Dental caries is one of the most prevalent oral diseases caused by bacterial infections and is characterized by the progressive destruction of tooth structures resulting from continuous enamel and dentin demineralization [1]. Among various cariogenic microorganisms, *Streptococcus mutans* (*S. mutans*) is considered the primary pathogen responsible for dental caries [2]. This bacterium produces acids through the fermentation of dietary sucrose, leading to biofilm formation on tooth surfaces and subsequent enamel demineralization. Thus, strategies aimed at inhibiting bacterial activity and preventing enamel demineralization are crucial for effective caries prevention in clinical practice.

Topical fluoride application is currently one of the most widely adopted methods for preventing dental caries owing to its capacity to enhance enamel acid resistance and promote remineralization [3, 4]. Fluoride is available in various formulations such as fluoride varnishes, mouth rinses, gels, and toothpaste [4]. Among these products, fluoride varnish, which typically contains 5% sodium fluoride (NaF), is clinically preferred because it consistently releases fluoride for 24–48 h after application, providing sustained enamel protection with minimal risk of toxicity. However, recent studies have raised concerns regarding fluoride toxicity and dental fluorosis due to frequent fluoride exposure, emphasizing the need for cautious and controlled use [5]. Therefore, a novel strategy that reduces the frequency of fluoride application while simultaneously providing additional antibacterial benefits is required. In particular, incorporating antibacterial agents into fluoride varnishes may enhance their clinical efficacy by effectively suppressing cariogenic bacterial activity [6].

Recently, a light-curable fluoride varnish (LCFV) has been introduced to address the limitations associated with conventional fluoride varnishes. Previous studies have demonstrated that LCFV forms a robust and uniform coating, ensuring sustained fluoride release, and consequently providing superior long-term protection against enamel demineralization [7, 8]. Furthermore, LCFV offers rapid polymerization, easy handling, and improved operability, making it particularly beneficial for orthodontic patients with poor oral hygiene compliance [5, 8]. A recent approach involving the incorporation of zwitterionic materials into LCFV has demonstrated reduced film thickness, potentially improving their clinical application. However, the stable incorporation of these materials requires complex surface grafting processes, which limit their practical implementation [8]. More recently, a LCFV containing bioactive glass 63 S was shown to promote enamel remineralization, highlighting the potential of bioactive additives to expand the therapeutic scope of these materials [9]. Collectively, these studies represent the most up-to-date advances in LCFV modification and emphasize the need for simpler and more practical antibacterial additives suitable for clinical integration.

Strontium (Sr) is known for its capacity to promote dentin remineralization and to provide desensitizing effects. Moreover, Sr ions exhibit substantial antibacterial properties by disrupting the bacterial cell membrane integrity and enzyme activity [10]. Phosphate-based glasses (PBGs), characterized by compositions similar to those of natural tooth and bone tissues, demonstrate excellent biocompatibility and continuously release calcium (Ca) and phosphate (P) ions, thereby facilitating effective remineralization [10]. This research highlights that strontium-containing phosphate-based glasses (Sr-PBGs) exhibit robust antibacterial and bioactive properties. Additionally, when incorporated into dental composite resins, Sr-PBG effectively inhibits *S. mutans* without adversely affecting mechanical properties, such as flexural strength and elastic modulus [10].

Although numerous antibacterial agents have been investigated for incorporation into dental materials, no previous studies have evaluated the antibacterial efficacy and physicochemical properties of LCFV-containing Sr-PBGs. Considering the excellent antibacterial and bioactive properties of Sr-PBG, its incorporation into LCFV is expected to concurrently enhance its antibacterial activity and enamel protection, addressing the limitations of conventional formulations.

Therefore, this study aimed to systematically investigate the antibacterial effects and physicochemical properties of LCFVs containing various concentrations (1.0, 5.0, and 10.0 wt%) of Sr-PBG. The null hypothesis tested in this study was that there would be no significant differences in antibacterial efficacy and physicochemical properties between Sr-PBG-containing LCFVs and control LCFVs without Sr-PBG.

## Materials and methods

### Glass preparation

Sr-PBG powder was synthesized by mixing phosphorus pentoxide (P₂O₅; 50 mol%), sodium oxide (Na₂O; 20 mol%), calcium oxide (CaO; 15 mol%), and strontium oxide (SrO; 15 mol%) (Sigma-Aldrich, St. Louis, MO, USA). The raw materials were homogenized in a high-speed mixer (Hauschild, Hamm, Germany) for 5 min to achieve uniform distribution. The mixture was then melted in an alumina crucible at 1100 °C for 1 h, followed by rapid cooling to room temperature at 25 ± 1 °C to form glass cullet. The solidified glass was ground manually using an alumina mortar and further pulverized under dry conditions using a planetary monomill (Pulverisette 7, FRITSCH, Idar-Oberstein, Germany) to obtain a fine Sr-PBG powder.

### Powder characterization

The characteristics of the Sr-PBG powder were verified by X-ray diffraction (XRD) using an Ultima IV diffractometer (Rigaku, Tokyo, Japan). The diffraction data were collected over a 2*θ* range of 10° to 80° at a scanning speed of 1°/min. To investigate the morphology of the experimental powders, small amounts of Sr-PBG powder were attached to metal stubs using double-sided carbon tape. The samples were then sputter-coated with platinum (Pt) under vacuum for 100 s at 20 mA. Scanning electron microscopy (SEM) (JEOL-7800 F, JEOL Ltd., Tokyo, Japan) coupled with energy-dispersive X-ray spectroscopy (EDX) was performed at 2000 × magnification for elemental analysis. The particle size of the Sr-PBG powder was measured using a particle size analyzer (ELS-Z1000, Otsuka Electronics, Tokyo, Japan). Distilled water was used as the dispersion medium, and the analysis was performed at a rotational speed of 2000 rpm to prevent the sedimentation of agglomerated particles.

### Specimen preparation

To prepare fluoride varnish specimens containing Sr-PBG, commercial fluoride varnish (3 M™ Clinpro™ XT Vanish Durable Fluoride Releasing Coating, 3 M ESPE, St. Paul, MN, USA), which consists of silane-treated glass, 2-hydroxyethyl methacrylate, water, (1-methylethylidene)bis[4,1-phenyleneoxy(2-hydroxy-3,1-propanediyl)] bismethacrylate, silane-treated silica, copolymer of acrylic and itaconic acids, calcium glycerophosphate, diphenyliodonium hexafluorophosphate, and ethyl acetate, was mixed with Sr-PBG powder at varying weight ratios of 0% (SrPBG0, control), 1.0% (SrPBG1), 5.0% (SrPBG5), and 10.0% (SrPBG10). Each mixture was homogenized using a high-speed mixer (SpeedMixer DAC 150/250, Hauschild & Co. KG, Hamm, Germany) at 1500 rpm for 2 min. The prepared mixture was slightly overfilled into a metal mold (10.0 mm in diameter and 1.0 mm in thickness) placed on a glass plate covered with a polyethylene film. Subsequently, another polyethylene film and a glass plate were placed on top, and the assembly was clamped securely together. Each specimen was then polymerized by irradiating both the top and bottom surfaces of the mold for 20 s with a light-curing unit (LDCL-30, DENTALL Co. LTD, Bucheon, Korea). After curing, the specimens were removed from the molds, and any excess cured material was polished using 2,000-grit silicon carbide abrasive paper (Deerfos, Incheon, Korea) to obtain uniformly shaped circular specimens (10.0 mm in diameter and 1.0 mm in thickness).

### Degree of conversion

The degree of conversion (DC) of each varnish formulation was assessed using Fourier-transform infrared spectroscopy (FT-IR; INVENIO, Bruker, Ettlingen, Germany). The samples were analyzed at two time points: immediately after mixing (NP, not polymerized) and after the completion of photopolymerization (P, polymerized). FT-IR analysis was conducted using a horizontal attenuated total reflectance unit containing a diamond crystal (2 mm in diameter). The analyzed site on each sample surface measured approximately 800 μm in diameter. Spectra were obtained between 1400 and 2000 cm⁻¹ at a resolution of 4 cm⁻¹, with two scans performed per second. The DC calculation involved measuring changes in the absorbance peak area ratio of the aliphatic methacrylate double bond (1638 cm⁻¹) to an aromatic carbon double bond (1608 cm⁻¹, used as an internal reference), comparing the uncured state to the cured state. The percentages of DC were calculated as follows:

$$\:DC\left(\%\right)=\left\{1-\frac{Abs\frac{C=C1638c{m}^{-1}\left(P\right)}{C=C1608c{m}^{-1}\left(P\right)}}{Abs\frac{C=C1638c{m}^{-1}\left(NP\right)}{C=C1608c{m}^{-1}\left(NP\right)}}\right\}\times\:100$$

### Film thickness

Film thickness was determined in accordance with ISO 4049:2019 (Dentistry—Polymer-based restorative materials). First, the initial thickness of two glass plates (area: 200 ± 25 mm², thickness: 5 mm each) was measured using a digital micrometer (Absolute Digimatic, Model 293–805; Mitutoyo Corp., Kanagawa, Japan). Immediately after mixing, 0.1 mL of each sample was placed at the center of one glass plate and covered with the other plate. Subsequently, a vertical load of (150 ± 2) N was applied to the center of the sample using a loading apparatus for 180 ± 10 s. After loading, the combined thicknesses of the two glass plates and the specimen were measured again using a micrometer. The film thickness was calculated as the difference between the combined thicknesses of the two glass plates with and without uncured varnish materials. The measurements were repeated six times per specimen, and the average value was recorded as the representative thickness.

### Contact angle

The contact angle was measured using a contact angle analyzer (SmartDrop Plus, Femtobiomed, Sungnam, Gyeonggi, Korea) on the surface of disc-shaped specimens (diameter: 10.0 mm, thickness: 1.0 mm). Each specimen was placed horizontally on the sample stage of the analyzer, and a distilled water droplet (3 µL) was deposited onto the specimen surface. After 5 s, the contact angles were recorded. The measurements were repeated three times at randomly selected sites on each specimen surface, and the average of these measurements was calculated to represent the contact angle of each specimen.

### SEM observation

The surface morphologies of the disc specimens (diameter: 10.0 mm, thickness: 1.0 mm) were evaluated using SEM. The surface microstructures of the specimens were observed under a magnification of 2,000× at an accelerating voltage of 7.0 kV. SEM images were captured to examine and characterize the surface micromorphological features of the experimental specimens.

### Bacterial cultivation and evaluation of antibacterial activity against Streptococcus mutans

The bacterial strain used in this experiment was *S. mutans* (*S. mutans*, KCTC No. 3065; Korean Collection for Type Cultures, Jeonbuk, Korea). *S. mutans* was cultured using brain heart infusion (BHI; Becton Dickinson and Co., Sparks, MD, USA) medium without sucrose to evaluate the direct antibacterial effect of the specimens, independent of biofilm formation enhanced by sucrose. Initially, freeze-dried powdered *S. mutans* was inoculated into BHI broth and incubated at 36.5 °C for 48 h in an incubator (JSGI-150T, JSR, Gongju, Korea) for primary culture. The bacterial suspension obtained from this primary culture was diluted at a ratio of 1:2 with fresh BHI broth, redistributed, and incubated again at 36.5 °C for an additional 24 h for secondary culture. The bacterial concentration of the final cultured suspension was adjusted with additional BHI broth to achieve an optical density range of 0.4–0.6, measured by spectrophotometry (Biomate 3 S, Thermo Fisher Scientific, Madison, WI, USA) at 600 nm, which corresponds to the mid-logarithmic growth phase of *S. mutans* to ensure a metabolically active and standardized inoculum [11].

Prior to antibacterial testing, all disc-shaped specimens (diameter 10.0 mm; thickness 1.0 mm) were sterilized under UV light for 30 min on each side prior to antibacterial testing. The sterile specimens were then individually placed into the wells of a 24-well plate (SPL Life Science, Gyeonggi-do, Korea). One milliliter of the adjusted *S. mutans* suspension was dispensed into each well, and the specimens were completely immersed. As a negative control, wells containing bacterial suspensions without specimens were also prepared. The plates were then incubated at 36.5 °C for 24 and 48 h. After each incubation period, aliquots of 100 µL from each bacterial suspension were spread onto agar plates, followed by an additional incubation at 36.5 °C for 48 h. Finally, colony-forming units (CFUs) were counted manually using a magnifying glass to ensure accuracy, and the counts were cross-verified by two independent researchers. The average value was calculated and used for further analysis.

For the morphological observation of *S. mutans* attached to the surface of the experimental varnish, sterilized disc-shaped specimens (diameter: 10.0 mm, thickness: 1.0 mm) were individually positioned horizontally in 24-well plates. A bacterial suspension of *S. mutans* (OD_600_:0.5) was prepared, and 100 µL was carefully dispensed onto each specimen, ensuring that the suspension uniformly covered the entire specimen surface. The plates containing specimens were subsequently incubated at 37 ± 1 °C for 48 h. After incubation, the specimens were gently washed with distilled water for 10 s to remove loosely attached bacteria. Following washing, each specimen was fixed for 24 h at room temperature in Karnovsky’s fixative solution, comprising 2% paraformaldehyde, 2% glutaraldehyde, and 0.1 M phosphate buffer solution (PBS). The fixed specimens were rinsed with 0.1 M PBS for 30 min and then fixed with 1% osmium tetroxide for 2 h. After secondary fixation, the specimens were sequentially dehydrated using an ethanol gradient (50–100%) and subsequently dried using a critical point dryer (Leica EM CPD300, Leica, Wien, Austria) for 2 h. Finally, the dried specimens were sputter-coated with platinum using an ion sputter coater (Leica EM ACE600, Leica, Wien, Austria), and the morphology of *S. mutans* adhered to the specimens was observed under a field emission scanning electron microscope (FE-SEM; Merlin, Carl Zeiss, Oberkochen, Germany) operated at an accelerating voltage of 5.0 kV.

### Ion release

To evaluate the ion release, disc-shaped specimens (diameter: 10.0 mm, thickness: 1.0 mm) were individually immersed in 5 mL of distilled water within separate conical tubes. These tubes were then incubated at 37 ± 1 °C in a water bath for 48 h. The volume of 5 mL was chosen to satisfy the minimum sample requirement for ion analysis while maintaining consistency with the antibacterial assay conditions in terms of specimen geometry, temperature, and incubation time. After the designated incubation period, the specimens were removed, and the concentrations of Ca, P, and Sr ions released into distilled water were quantitatively determined using an inductively coupled plasma triple quadrupole mass spectrometer (Agilent 8900, Agilent Technologies, CA, USA). Each eluate was analyzed in triplicate, and the average value was calculated as the representative ion release for each specimen.

### Statistical analysis

Statistical analysis of the data obtained in this study was performed using IBM SPSS Statistics for Windows (version 28.0; IBM Corp., Armonk, NY, USA). To determine significant differences according to the concentration of each variable, a one-way analysis of variance was conducted, followed by Tukey’s post-hoc test for multiple comparisons between the groups. The level of statistical significance was set at *p* < 0.05.

## Results

### Powder characterization

**Fig. 1:**
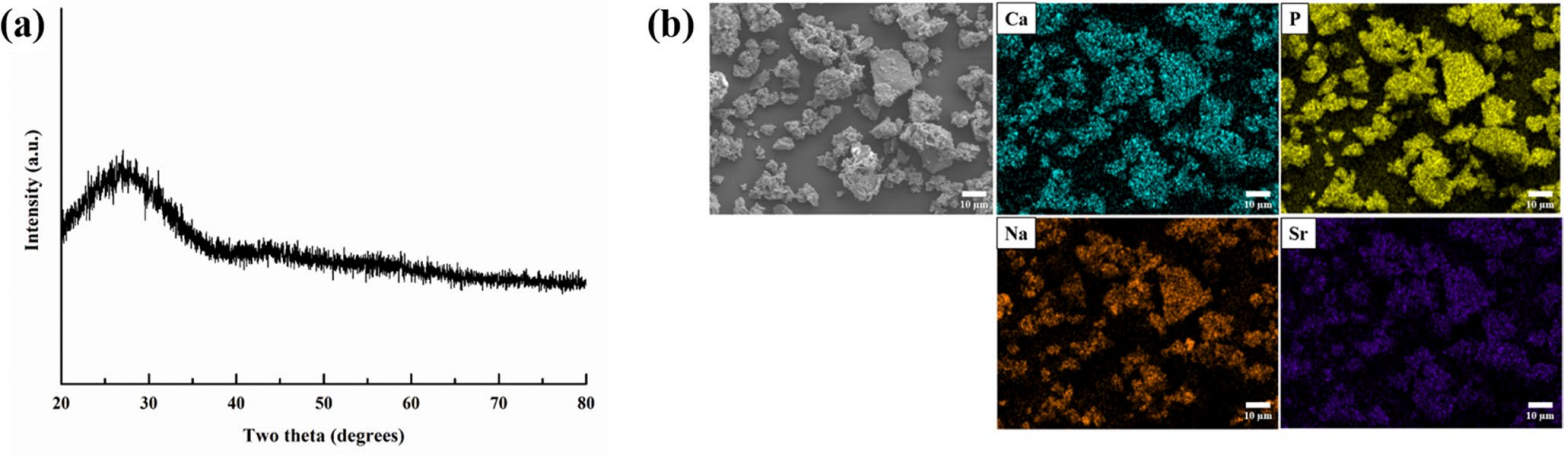
Characterization of Sr-PBG powder. (**a**) X-ray diffraction pattern of the Sr-PBG powder. (**b**) Scanning electron microscopy image and corresponding energy-dispersive X-ray spectroscopy (EDX) elemental mapping images. The EDX maps illustrate a uniform distribution of calcium (Ca, light blue), phosphorus (P, yellow), sodium (Na, orange), and strontium (Sr, purple) throughout the sample

The XRD pattern of the Sr-PBG powder is presented in Fig. 1a, exhibiting a broad diffraction feature without distinct sharp peaks, confirming its amorphous structure. Figure 1b shows the morphology of Sr-PBG particles. The particles exhibited irregular shapes and tended to aggregate into clusters. The corresponding EDX elemental mapping images demonstrated a homogeneous distribution of Ca, P, sodium (Na), and Sr throughout the particles. Particle size analysis indicated that the average particle diameter of the Sr-PBG powder was 774.8 ± 7.1 nm.

### Degree of conversion

**Fig. 2:**
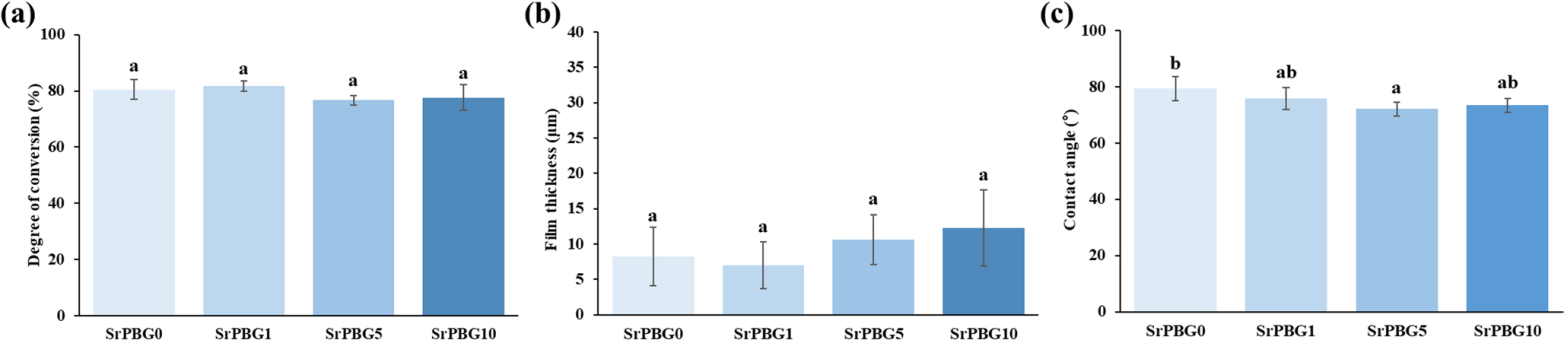
(**a**) Degree of conversion, (**b**) film thickness, and (**c**) contact angle of light-curable fluoride varnishes: control group without Sr-PBG (SrPBG0), and experimental groups containing 1 wt% (SrPBG1), 5 wt% (SrPBG5), and 10 wt% (SrPBG10). Data are presented as mean ± SD (*n* = 6 per group). Statistical analysis was performed using one-way ANOVA followed by Tukey’s post hoc test. Different lowercase letters above the bars indicate significant differences among groups (*p* < 0.05)

The degree of conversion (%) was 80.48 ± 3.55% for the control group (SrPBG0) without Sr-PBG, and 81.73 ± 1.71%, 76.70 ± 1.63%, and 77.67 ± 4.45% for the experimental groups SrPBG1, SrPBG5, and SrPBG10, respectively. Statistical analysis indicated no significant difference between the Sr-PBG-treated and control groups (*p* > 0.05). Furthermore, increasing the Sr-PBG concentration did not significantly influence the degree of conversion (*p* > 0.05) (Fig. 2a).

### Film thickness

The measured film thickness values were 8.20 ± 4.14 μm for the control group (SrPBG0), and 7.00 ± 3.34 μm, 10.66 ± 3.51 μm, and 12.25 ± 5.37 μm for the experimental groups SrPBG1, SrPBG5, and SrPBG10, respectively. Statistical analysis revealed no significant differences between the Sr-PBG-treated and control groups (*P* > 0.05). Furthermore, increasing the concentration of Sr-PBG did not significantly affect the film thickness (*p* > 0.05) (Fig. 2b).

### Contact angle

The measured contact angles were 79.36 ± 4.32° for the control group (SrPBG0), and 75.77 ± 3.84°, 72.15 ± 2.46°, and 73.48 ± 2.47° for the experimental groups SrPBG1, SrPBG5, and SrPBG10, respectively. Statistical analysis revealed that the control group (SrPBG0) did not significantly differ from the SrPBG1 and SrPBG10 groups (*p* > 0.05); however, there was a significant difference compared with the SrPBG5 group (*p* < 0.05). Moreover, variations in the Sr-PBG concentration had no significant effect on the contact angle (*p* > 0.05) (Fig. 2c).

### SEM

**Fig. 3:**
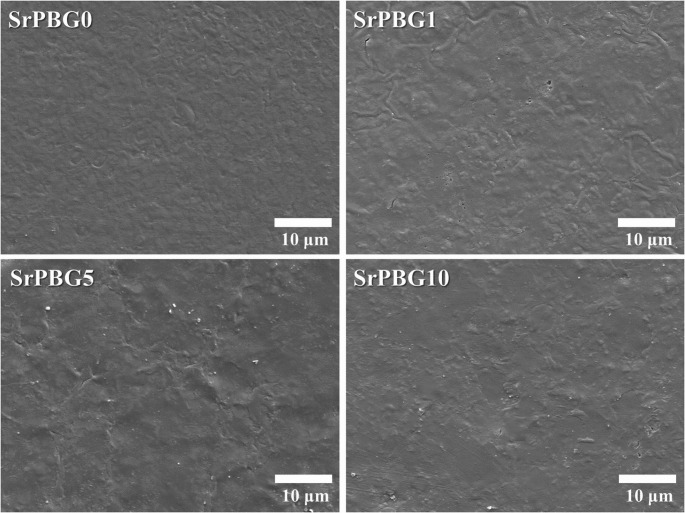
shows the SEM images of the specimen surfaces. Both the control and experimental groups appeared smooth under SEM observation. With increasing Sr-PBG concentration, localized particle agglomerates were observed on the varnish surfaces, which gave a slightly uneven appearance

Figure 3 Scanning electron microscopy images illustrate the surface morphologies of the light-curable fluoride varnishes with different Sr-PBG concentrations. SrPBG0 represents the control group without Sr-PBG, whereas SrPBG1, SrPBG5, and SrPBG10 indicate varnishes with increasing concentrations of Sr-PBG. Scale bars represent 10 μm.

### Evaluation of antibacterial effect

**Fig. 4:**
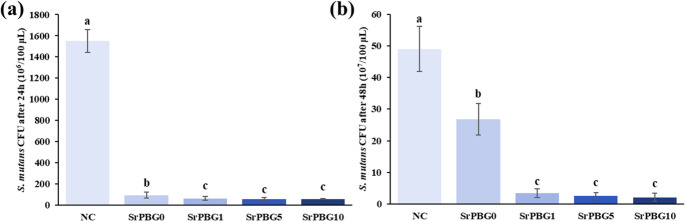
Antibacterial activity of light-curable fluoride varnishes containing varying concentrations of Sr-PBG against *S. mutans*. (**a**) CFU counts after 24 h of incubation. (**b**) CFU counts after 48 h of incubation. Data are shown as mean ± SD (*n* = 6 per group). Statistical analysis was performed using one-way ANOVA followed by Tukey’s post hoc test. Different lowercase letters indicate statistically significant differences between groups (*p* < 0.05)

The CFU counts after 24 h of incubation were as follows: negative control (NC), 1551.00 ± 106.10 × 10⁶/100 µL; SrPBG0, 92.67 ± 30.03 × 10⁶/100 µL; SrPBG1, 62.00 ± 15.78 × 10⁶/100 µL; SrPBG5, 55.00 ± 15.05 × 10⁶/100 µL, and SrPBG10, 57.00 ± 4.24 × 10⁶/100 µL. After 48 h, CFU values were NC, 49.00 ± 7.10 × 10^7^/100 µL; SrPBG0, 26.75 ± 4.99 × 10^7^/100 µL, SrPBG1, 3.50 ± 1.37 × 10^7^/100 µL, SrPBG5, 2.66 ± 1.03 × 10^7^/100 µL, and SrPBG10, 2.14 ± 1.34 × 10^7^/100 µL (Fig. 4). All Sr-PBG-containing groups showed significantly lower CFU counts than the SrPBG0 group (*p* < 0.05). Notably, after 48 h, the SrPBG1, SrPBG5, and SrPBG10 groups exhibited markedly reduced CFU values compared to SrPBG0, highlighting that the difference in antibacterial effectiveness between the control and Sr-PBG groups became more pronounced with prolonged incubation. Meanwhile, no statistically significant differences were observed among the SrPBG1, SrPBG5, and SrPBG10 groups at either the 24–48 h incubation periods (*p* > 0.05).

### SEM analysis of bacterial adhesion on specimen surfaces after 48 h incubation with S. mutans

**Fig. 5:**
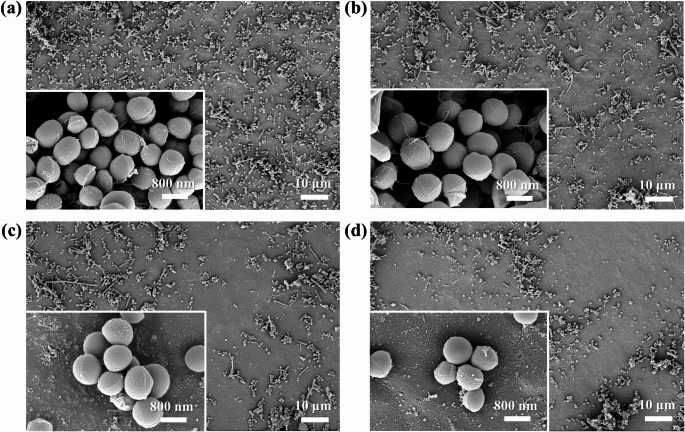
SEM images illustrating the adherence of *S. mutans* to specimen surfaces after 48 h of incubation. (**a**) SrPBG0 (control without Sr-PBG); (**b**) SrPBG1; (**c**) SrPBG5; (**d**) SrPBG10. Main images were captured at × 1,000 magnification (scale bar = 10 μm), and inset images were captured at ×20,000 magnification (scale bar = 800 nm)

As shown in Fig. 5, S. *mutans* adhered extensively to the surface of the SrPBG0 specimen, exhibiting the highest bacterial attachment density among all tested groups. In contrast, specimens containing Sr-PBG showed reduced bacterial adherence compared to SrPBG0. Notably, SrPBG10 displayed the lowest density of bacterial attachment among all Sr-PBG-containing groups.

### Ion release analysis

**Fig. 6:**
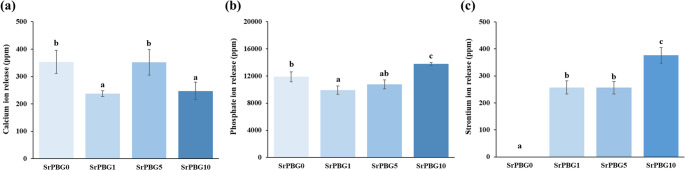
Ion release from the control group (SrPBG0) and experimental groups (SrPBG1, SrPBG5, SrPBG10) after immersion in distilled water for 48 h: (**a**) Ca ion release, (**b**) P ion release, and (**c**) Sr ion release. Data are presented as mean ± SD (*n* = 6 per group). Statistical analysis was performed using one-way ANOVA followed by Tukey’s post hoc test. Different lowercase letters above the error bars indicate statistically significant differences among the groups (*p* < 0.05)

The amount of released Ca ions was measured as 353.52 ± 42.20 ppm for SrPBG0, 237.79 ± 10.35 ppm for SrPBG1, 351.86 ± 46.79 ppm for SrPBG5, and 247.07 ± 32.23 ppm for SrPBG10. Statistical analysis revealed significant differences between the groups (*p* < 0.05) (Fig. 6a); however, no increase in Ca ion release associated with increasing SrPBG concentration.

The released P ions were 11878.90 ± 717.79 ppm for SrPBG0, 9903.64 ± 612.82 ppm for SrPBG1, 10752.94 ± 666.49 ppm for SrPBG5, and 13767.18 ± 217.57 ppm for SrPBG10. Statistically significant differences were observed among the groups, with the P ion release significantly increasing as the SrPBG concentration increased (*p* < 0.05) (Fig. 6b).

The release of Sr ions was not detected in the SrPBG0 group (0 ppm), while Sr ion concentrations were measured as 257.07 ± 24.01 ppm for SrPBG1, 256.27 ± 22.83 ppm for SrPBG5, and 376.25 ± 28.85 ppm for SrPBG10. Statistical analysis indicated significant differences among the groups, demonstrating that Sr ion release increased significantly in response to higher SrPBG concentrations (*p* < 0.05) (Fig. 6c).

## Discussion

Fluoride varnishes, which are widely used in clinical dentistry, continuously release fluoride ions onto tooth surfaces, promote enamel remineralization, and increase resistance to acid challenges, thus effectively preventing the progression of dental caries [12]. However, conventional fluoride varnishes lack direct antibacterial activity against cariogenic microorganisms, necessitating repeated clinical applications to maintain caries prevention [13]. Such repetitive applications are not only technique-sensitive but also impose significant economic burdens at both the individual and community levels [5]. Therefore, there is a clinical need for an innovative approach that incorporates antibacterial agents into fluoride varnishes while maintaining their favorable physicochemical properties. To address this need, this study aimed to evaluate the antibacterial effects against *S. mutans* and assess the physicochemical properties of an LCFV containing varying concentrations of Sr-PBG.

Fluoride has been reported to prevent enamel demineralization caused by acid production by *S. mutans* and to reduce bacterial counts in the oral cavity. However, fluoride varnishes applied alone exhibit minimal antibacterial activity against *S. mutans*, prompting recommendations for their combined use with other antibacterial agents [14]. Recent studies have reported that strontium ions can penetrate bacterial cells, disrupt essential biological processes, such as nucleic acid synthesis, protein production, and enzyme activity, and interfere with intracellular ion exchange mechanisms, thereby inhibiting bacterial viability and proliferation. Incorporation of Sr-PBG into dental materials, including composites and acrylic resins, has been shown to impart antibacterial properties while maintaining favorable mechanical and physicochemical performance [10, 15]. To our knowledge, the present study is the first to investigate the antibacterial effects of Sr-PBG within fluoride varnishes. In this study, Sr-PBG was incorporated up to 10 wt% to maintain the physicochemical integrity of the varnish, as higher loadings can compromise material stability. Lower to moderate levels were considered sufficient to provide antibacterial benefits while preserving the original properties of the varnish. Our findings demonstrated that fluoride varnishes containing Sr-PBG significantly reduced CFU counts of *S. mutans* at both 24 h and 48 h compared with the control, with more pronounced effects after 48 h. However, no significant differences were observed among Sr-PBG concentrations, suggesting that the antibacterial effect may plateau beyond a threshold level or be influenced by biological variability and particle distribution. A previous study on chlorhexidine-containing fluoride varnish reported strong initial activity against *S. mutans* and *S. sobrinus* that declined markedly after 48 h [16]. In contrast, the present varnishes maintained antibacterial activity over 48 h, likely due to the sustained-release properties of Sr-PBG. SEM analysis further supported these results, demonstrating reduced bacterial adhesion on varnishes containing Sr-PBG compared with the control group. Although previous studies have reported reduced bacterial adhesion by incorporating zwitterionic materials into LCFV, this approach primarily inhibited adhesion without directly affecting bacterial viability [8]. In contrast, Sr-PBG exhibited reduced bacterial adhesion and direct antibacterial activity, indicating its superior clinical potential. SEM images confirmed extensive bacterial colonization in the control group, whereas significantly reduced bacterial adhesion was observed in all Sr-PBG groups, with the lowest adhesion observed in the 10 wt% group. Thus, the null hypothesis of no significant antibacterial difference between the Sr-PBG-containing and control varnishes was rejected. Overall, these results suggest that fluoride varnishes incorporating Sr-PBG exhibit enhanced antibacterial performance compared with conventional fluoride varnishes, supporting their clinical utility as effective caries-preventive materials.

A low DC in dental materials can adversely affect their durability, as incomplete polymerization results in residual unreacted monomers that can dissolve or degrade in a moist oral environment, potentially compromising the structural integrity of the material [17]. Common methods used to evaluate the DC of dental materials include Fourier transform infrared (FT-IR) spectroscopy, microhardness measurements [18], and Raman spectroscopy. Among these techniques, FT-IR is recognized as the standard method for evaluating polymerization in dental materials because of its nondestructive nature, rapid analysis time, high accuracy with minimal sample requirements, and practical advantages over Raman spectroscopy [19]. In this study, FT-IR spectroscopy was used to determine the influence of Sr-PBG incorporation on the DC of LCFV and to identify the optimal Sr-PBG concentration. The results demonstrated no significant differences in DC between the Sr-PBG-containing groups and the control group, and an increase in Sr-PBG concentration did not significantly affect DC (*p* >0.05). These findings suggest that incorporation of Sr-PBG did not adversely affect polymerization and that the tested varnishes maintained stable physicochemical properties (film thickness, contact angle) under the conditions evaluated, which may be indicative of favorable performance in the oral environment.

An increased film thickness of dental materials can negatively affect intraoral adaptation and potentially cause patient discomfort. Conversely, an excessively thin coating may degrade rapidly due to oral factors such as food intake, saliva, and tooth brushing, thereby limiting the sustained release of fluoride [20, 21]. According to previous studies, fluoride-containing materials must form a dense and stable protective barrier on the enamel surface that can resist erosive acids to ensure effective and long-term enamel protection [22]. Therefore, in the present study, the film thickness was evaluated to confirm whether the addition of Sr-PBG maintained a film thickness similar to that of conventional fluoride varnishes. A previous study investigated film thickness by incorporating zwitterionic powders into LCFV at a 3 wt% ratio and measuring the thickness with a digital micrometer following ISO 4049 [8]. They reported a significant reduction in film thickness for the zwitterionic-containing group compared to the control (median, 10.0 μm; IQR, 3 μm). However, this reduced thickness may adversely affect material durability and lead to rapid abrasion from the tooth surfaces. In this study, the film thickness measurements were performed according to the method described by ISO 4049 [23]. Although ISO 4049 was originally established for dental resin cements and coating materials, its method of measuring film thickness under a 150-N load was considered appropriate for accurately assessing fluoride varnish thickness. According to the results obtained via ISO 4049 methodology, the film thickness values were 8.20 ± 4.14 μm for the control group and 7.00 ± 3.34 μm, 10.66 ± 3.51 μm, and 12.25 ± 5.37 μm for SrPBG1, SrPBG5, and SrPBG10 groups, respectively. Statistical analysis revealed no significant differences in film thickness between the Sr-PBG-containing and control groups (*p* >0.05). Additionally, increasing the Sr-PBG concentration did not significantly affect the film thickness (*p* >0.05). Thus, the findings of this study indicate that incorporating up to 10 wt% Sr-PBG into fluoride varnishes maintains a film thickness comparable to that of conventional varnishes, suggesting that Sr-PBG addition did not adversely affect the film-forming ability of the varnish. The absence of a concentration-dependent pattern may be attributable to minimal viscosity variations among the groups, which were insufficient to influence film formation under the standardized ISO 4049 testing conditions. Further rheological investigations are required to clarify the influence of Sr-PBG concentration on varnish flow behavior and film thickness.

Contact angle is widely used to evaluate the hydrophilicity or hydrophobicity of material surfaces by measuring the wettability of liquids on solid substrates [24]. In particular, fluoride varnishes applied to tooth enamel must exhibit sufficient adhesion and stability on tooth surfaces to enable prolonged fluoride ion release and effective enamel protection; these properties can be indirectly assessed through contact angle measurements [15]. Materials with lower contact angles have greater hydrophilicity, which typically results in enhanced adhesion to tooth surfaces [25]. A previous study investigating self-curing acrylic resins containing Sr-PBG reported no statistically significant changes in the contact angles with increasing Sr-PBG concentrations (*p* >0.05) [15]. In this study, the influence of Sr-PBG on the contact angle of fluoride varnishes was assessed. The results showed no significant differences between the control, SrPBG1, and SrPBG10 groups (*p* >0.05), whereas the SrPBG5 group exhibited a significantly lower contact angle than the control group (*p* < 0.05). The isolated difference observed in the SrPBG5 group is unlikely to reflect a systematic concentration-dependent effect and may instead be attributable to surface heterogeneity or experimental variability. Nevertheless, there was no overall trend of significant contact angle changes corresponding to increased Sr-PBG concentrations (*p* >0.05). Thus, the current findings are consistent with those of previous research, indicating that, in addition to minor differences at specific concentrations, the contact angle values generally remained unaffected by varying concentrations of Sr-PBG. Consequently, the addition of Sr-PBG to the fluoride varnishes did not appear to significantly alter the intrinsic surface characteristics of the varnish, maintaining its inherent physicochemical properties.

SEM analysis was conducted to examine the surface morphology and dispersion of Sr-PBG particles in fluoride varnishes with varying concentrations of Sr-PBG. SEM observations revealed that both the control and Sr-PBG-containing groups generally exhibited smooth and homogeneous surfaces. However, with increasing concentrations of Sr-PBG, slight particle agglomeration was observed in localized regions. Previous studies have reported that particle agglomeration can lead to stress concentrations within materials, potentially reducing flexural strength [10]. Additionally, increased surface roughness due to particle agglomeration may facilitate bacterial adhesion and plaque accumulation [26, 27]. In a similar previous study involving heat-polymerized acrylic resins incorporating silver zeolite nanoparticles, SEM analysis showed that increased nanoparticle concentrations led to higher surface roughness, characterized by microscale elevations and depressions [28]. In the present study, no major cracks or defects were observed in the SEM images, although localized agglomeration appeared at higher Sr-PBG concentrations (Fig. 3). Therefore, future studies should consider improved dispersion techniques and optimization of Sr-PBG content to minimize agglomeration and ensure consistent surface properties and antibacterial efficacy.

Strontium can substitute for Ca ions in the hydroxyapatite lattice due to its chemical similarity, thereby enhancing crystal stability, increasing resistance to acidic conditions, and promoting fluoride ion binding under neutral pH, ultimately facilitating enamel remineralization [29]. In addition, the continuous release of Sr ions has been reported to stimulate biological activity by influencing cellular processes such as osteoblast differentiation and mineral metabolism, providing further benefits in the oral environment. Meanwhile, phosphate ions present in saliva can interact with calcium ions to form amorphous calcium phosphate, which subsequently transforms into stable crystalline phases such as dicalcium phosphate dihydrate, octacalcium phosphate, and calcium-deficient hydroxyapatite, further contributing to the remineralization process [30].

In this study, fluoride varnishes containing Sr-PBG showed concentration-dependent increases in Sr and P ion release. In contrast to Sr and P, which increased proportionally, Ca release displayed a non-linear pattern with a higher value at the medium concentration (SrPBG5). The Ca and P release observed in the control group (SrPBG0) can be attributed to the intrinsic composition of the commercial varnish (Clinpro™ XT Vanish), as its formulation includes calcium glycerophosphate, which provides a baseline source of these ions. In addition, this behavior may be related to partial substitution of Ca by Sr within the glass network, which can alter bonding and solubility, leading to variable Ca elution. Similar non-linear Ca release profiles have been reported in Sr-doped bioactive glasses, where Sr incorporation modifies the glass network and affects Ca ion release [31]. Unlike fluoride varnishes with zwitterionic additives, which exhibited negligible Ca and P release [8], the incorporation of Sr-PBG is expected to enhance remineralization potential by supplying Sr together with Ca and P ions. The sustained ion release characteristics of Sr-PBG-containing varnish may contribute to favorable performance in the oral environment [8, 29].

Ion release was measured at a single time point of 48 h to match the incubation period of the antibacterial assays, enabling direct comparison between ion concentrations and antibacterial outcomes under identical conditions. While this approach is appropriate for evaluating short-term antibacterial relevance, it does not capture long-term release kinetics. Considering that fluoride varnishes are generally cleared from the oral cavity within a short period, extended measurements were beyond the scope of this study. Nevertheless, residual material may continue to influence ion release, and future studies should investigate longer-term release profiles. Although fluoride release is a critical feature of fluoride varnishes, it was not evaluated in this study because its behavior has been extensively documented in previous literature. Instead, we focused on Sr-PBG-derived ions (Sr, Ca, and P) to clarify their specific contributions to antibacterial activity and remineralization potential. Future studies should incorporate fluoride release measurements together with Sr, Ca, and P analysis to provide a more comprehensive assessment of material performance.

This study systematically evaluated the antibacterial efficacy and physicochemical properties of fluoride varnishes containing Sr-PBG. However, it had several limitations. First, the antibacterial evaluation was conducted using only a single bacterial species, *S. mutans*, a major cariogenic pathogen. Thus, this evaluation may not fully reflect the complex multispecies biofilm environment present in the oral cavity. Second, physicochemical analyses were performed under static immersion conditions, whereas the actual oral environment involves dynamic interactions, including continuous salivary flow, dietary factors, and mechanical abrasion through tooth brushing and contact with oral tissues [32]. Therefore, further research is warranted to assess the long-term antibacterial efficacy and physicochemical stability under dynamic conditions, as well as to evaluate the antibacterial activity against multispecies biofilms. Such additional studies would help establish the clinical applicability and effectiveness of Sr-PBG-containing fluoride varnishes.

Despite these limitations, the present findings demonstrate that incorporating Sr-PBG into fluoride varnishes preserved their favorable physicochemical properties (degree of conversion, film thickness, contact angle, and SEM characteristics), irrespective of the Sr-PBG concentration, while simultaneously providing significant antibacterial activity against *S. mutans*. Consequently, the null hypothesis that there would be no significant differences in antibacterial efficacy or physicochemical properties between the Sr-PBG-containing varnishes and the control group was rejected. In conclusion, the newly developed fluoride varnish incorporating Sr-PBG not only maintains the desirable physicochemical properties of conventional fluoride varnishes while providing additional antibacterial activity. The release of Sr, Ca, and P ions suggests a potential to support remineralization, indicating that Sr-PBG–containing varnishes may serve as promising adjunctive materials for caries prevention. Further studies are required to directly verify their remineralization capacity and long-term clinical effectiveness.

## Conclusions

This study evaluated the antibacterial efficacy and physicochemical properties of LCFV containing Sr-PBG. Incorporation of ≥ 1 wt% Sr-PBG significantly enhanced antibacterial activity against *S. mutans*, while up to 10 wt% did not adversely affect polymerization, film thickness, contact angle, or surface morphology. Higher concentrations also promoted continuous Sr and P ion release, which may support remineralization. Overall, Sr-PBG-containing LCFV preserved the properties of conventional varnishes while providing additional antibacterial activity and ion release, suggesting potential as an adjunctive approach for caries prevention.

## Data Availability

The data that support the findings of this study are available from the corresponding author upon reasonable request.
